# Ethnicity and association with disease manifestations and mortality in Behçet’s disease

**DOI:** 10.1186/1750-1172-9-42

**Published:** 2014-03-27

**Authors:** Lea Savey, Mathieu Resche-Rigon, Bertrand Wechsler, Cloé Comarmond, Jean Charles Piette, Patrice Cacoub, David Saadoun

**Affiliations:** 1 Department of Internal medicine and Clinical Immunology, Centre de référence des maladies autoimmunes et systémiques rares, AP-HP, Hôpital Pitié-Salpétrière, Paris, France; 2 Pierre et Marie Curie-Paris VI University, DHU I2B, Inflammation, Immunopathologie, Biothérapie, Université Pierre et Marie Curie, Paris VI, France; 3 Department of Biostatistics and Medical Data Processing; INSERM U717, Hôpital Saint-Louis, Paris, France

**Keywords:** Behçet’s disease, Mortality, Vasculitis, Ethnic origin

## Abstract

**Background:**

Behçet’s disease (BD) significantly increases morbidity and mortality. BD mainly affects young adults with a peculiar geographical distribution. It has been suggested that BD varies in its phenotypic expression in different ethnic groups.

**Methods:**

We investigated potential ethnicity-related differences relative to phenotype and prognosis of BD patients in a French multiethnic country. We included 769 consecutive patients fulfilling the international criteria of classification for BD, in the 3 largest ethnic groups of our cohort [European (n = 369), North African (n = 350) and sub Saharan African (n = 50)]. Factors that affect prognosis were assessed by multivariate analysis.

**Results:**

535 (69.6%) patients were male and the median (IQR) age at diagnosis was of 30.9 (24.9-37.2) years. Sub Saharan African BD patients had a higher frequency of CNS involvement (48% vs 32.3% vs 29.5%, p = 0 .035), a higher rate of death (12% vs 6% vs 3.5%, p = 0.029) and a lower frequency of HLA B51 allele (29.4% vs 49.2% vs 55.8%, p = 0.009) compared to those from North Africa and Europe, respectively. Multivariate analysis showed that male gender (HR: 5.01, CI: 1.51-16.65), cardiovascular involvement (HR: 2.24, CI: 1.15-4.36), and sub Saharan African origin (HR 2.62 (0.98-6.97) were independently associated with mortality. The 15-year mortality rate was of 19%, 9% and 6% in sub Saharan African, North African and European BD patients, respectively (p = 0.015).

**Conclusion:**

We reported ethnicity-related differences with respect to phenotype of BD. Sub Saharan Africans patients exhibited a worse prognosis.

## 

Behçet’s disease (BD) or Adamantiades-Behcet's Disease is a chronic, relapsing, vasculitis of unknown aetiology characterized by mucocutaneous, ocular, articular, vascular, gastrointestinal, and central nervous system manifestations [1]. BD significantly increases morbidity and mortality. BD mainly affects young adults with a peculiar geographical distribution, also named the “silk-road” – corresponding to the ancient route between the Mediterranean, the Middle East and the Far East. With the exception of oral aphthosis, BD is characterized by considerable phenotypic variation. Over the last 30 years, a substantial body of knowledge has accumulated supporting a strong genetic underpinning in BD of the MHC-related allele HLA-B5, which was later more specifically linked to its predominant suballele HLA-B51 [2]. It has been suggested that the disease varies in its phenotypic expression in different races and in different countries. Both environmental and genetic factors play a role in the aetiology of the condition [3]. For instance, BD patients from Asia exhibit a higher frequency of gastrointestinal involvement compared to those from the Mediterranean basin [4]. It has been reported that there is a greater risk of ocular involvement in patients in Japan or Iran and a lower risk of genital ulceration in most non-western countries [3]. However, most of the evidence supporting these propositions arises from observational case series, which are subject of many sources of bias. Moreover, to our knowledge no data are available regarding BD in sub Saharan African population. The present study investigated potential ethnicity-related differences relative to phenotype and prognosis of BD patients in a French multiethnic country. To this aim, we compared the main features of BD, the outcome and the factors associated with mortality in the 3 largest ethnic groups of our cohort (i.e. European, North African and sub Saharan African patients).

### Patients and method

#### Patients

Clinical records of 769 consecutive patients fulfilling the international criteria of classification for BD [5] were analyzed. All patients were referred to and regularly followed in the Internal medicine department of the Pitié-Salpétrière university hospital, Paris, France between 1974 and 2010. For each patient, the following data were collected: age at diagnosis of BD, gender, date of criteria for BD, geographic origin, main features of BD including mucocutaneous manifestations, ocular lesions, rheumatologic manifestations (athralgia, arthritis), neurologic involvement and/or cardiovascular involvement (venous, arterial and cardiac lesions). The number of BD flare, treatment, outcome and causes of death were recorded. 568 patients with BD were investigated for HLA B5 typing. The 3 largest ethnic groups originated from Europe (n = 369), North Africa (n = 350) and sub Saharan Africa (n = 50). Ethnicity was defined as the country of origin of the patient’s parents and grandparents and subjects were classified into 1 of the following defined ethnic groups: European, North African (Moroccan, Algerian, Tunisian, and Egyptian) and sub Saharan African. We considered only the European, North African and sub Saharan African patients, given the relatively small number of patients in the other racial groups. The diagnosis of neuro-BD was based on objective neurological symptoms not explained by any other known disease or therapy associated with neuroimaging findings suggestive of BD related central nervous system (CNS) involvement and/or cerebrospinal fluid (CSF) findings showing aseptic inflammation. Patients with non-parenchymal CNS involvement, or who did not show any abnormality on neurological examination or those without evidence of objective neurological involvement (i.e. only headache or dizziness) were excluded. The diagnosis of cardiovascular involvement included venous, arterial and cardiac lesions related to BD. Diagnosis of venous thrombosis was based on imaging data: venous doppler sonography, phlebography, cavography, computed tomography angiography and/or angio-magnetic resonance imaging (MRI). Diagnosis of arterial manifestations was based on imaging data: doppler sonography, arteriography, computed tomography angiography and/or angio-MRI. Cardiac involvement (pericarditis, myocardiopathy, myocardial infarction, endomyocardial fibrosis, thrombosis, valve insufficiency and/or stenosis) was considered to be related to BD if it was contemporaneous with BD flares and if other known causes of cardiac disease had been excluded.

#### Literature review

We systematically screened the medical literature via PubMed using the following keywords: “Behcet’s disease”, “Behcet’s syndrome”, “ethnology”, “ethnicity”, and “epidemiology”. We only analyzed cases series published after 1988 in English or in French. Studies focusing on ethnicity related phenotype or prognosis differences in adults BD patients were included. However, it should be acknowledged that these different studies have different approaches in case findings. Some studies were based on epidemiological estimates while others describe patients from tertiary referral centres and the latter were based on questionnaire or population samples. The methodological differences can be substantial and might affect the outcome of different cohorts.

#### Statistical analysis

Data are summarized as frequencies and percentages for categorical variables. Quantitative variables are presented as medians and 25th and 75th percentiles. Patients characteristics were compared using χ2 tests (or χ2 tests with Yates’s correction or Fisher’s exact test when required) for categorical data and Kruskal-Wallis rank sum tests for quantitative data. Survival was estimated using the Kaplan-Meier method. Patients were censored at the date of their last visit. Factors associated with the occurrence of death were assessed using a Cox proportional hazard model. Proportional hazard assumptions were checked. Hazard-ratios (HR) with their ninety-five percent confidence intervals (95% CI) are presented as a measure of association. All factors with P-value lower than 0.05 in the univariable analysis were included in a multiple Cox proportional hazard model. A model selection based on p-value was then performed. All tests were two-sided at the 0.05 significance level. Analyses were performed using R 2.15.1 statistical package. This study has been carried out in compliance with the Helsinki declaration.

### Results

#### Characteristics of BD patients

The main features of the 769 BD patients are summarized in Table 1. The median (IQR) age at diagnosis was 30.9 (24.9-37.2) years with 535 (69.6%) male. Patients originated from Europe (47.9%), North Africa (45.5%) and sub Saharan Africa (6.5%). The HLA B5 typing was positive in 51.4% of cases. Main clinical signs of BD included eye involvement (65.9%), genital ulcerations (60.4%), articular involvement (48.6%), cardiovascular involvement (42.6%) and central nervous system involvement (31.7%). The median number of BD’s flare was of 3 (2–5).

**Table 1:** Characteristics of Behçet’s disease according to ethnic origin*

**Parameters**	**Europe**	**Sub saharan Africa**	**North Africa**	**p-value**
	**(n = 369)**	**(n = 50)**	**(n = 350)**	
Age at diagnosis (years)	30.6 [24.9; 36.9]	32.21 [24.9; 40.7]	30.59 [24.5; 37.2]	0.55
Time between first symptom to diagnosis of BD (years)	3.1 [0.2; 7.9]	1.9 [0.04; 7.6]	1.9 [0.04; 7.6]	0.059
Maler gender	193 (52.3)	39 (78)	273 (78)	<0.0001
Genital ulceration	217 (58.81)	31 (62)	217 (62)	0.66
Articular involvement	188 (51.09)	23 (46)	163 (46.7)	0.47
Ocular involvement	245 (66.4)	26 (53.06)	236 (67.43)	0.14
CNS involvement	108 (29.51)	24 (48)	112 (32.28)	0.035
CV involvement	152 (41.19)	27 (54)	149 (42.57)	0.22
HLAB51	163/292 (55.82)	10/34 (29.41)	119/242 (49.17)	0.009
Number of BD flares	3 [2;5]	3 [2;4.5]	3 [2;5]	0.81
Immunosuppressants	178 (48.24)	26 (52)	206 (58.86)	0.016
Glucocorticosteroids	239 (64.77)	33 (66)	238 (68)	0.66
Anticoagulation	27 (7.32)	2 (4)	20 (5.71)	0.63
Death	13 (3.52)	6 (12)	21 (6)	0.029

*Except where indicated otherwise values are the median, IQR or n, percentage.

CNS, central nervous system; BD, Behçet’s disease; CV, cardiovascular.

The comparison between main characteristics according to ethnic origin of BD patients showed a higher frequency of male in sub Saharan and North African patients compared to those from Europe (78% and 78% vs 52.3%, respectively). The male/female ratio in the sub-Saharan and North African groups was of 3.5. There was a higher frequency of CNS involvement (48% vs 32.3% vs 29.5%) (p = 0.035), a higher rate of death (12% vs 6% vs 3.5%) (p = 0.029) and a lower frequency of HLA B51 allele (29.4% vs 49.2% vs 55.8%) (p = 0.009) in patients from sub Saharan Africa compared to those from North Africa and Europe, respectively. The time between first symptoms and diagnosis of BD was not significantly different according to ethnic groups (Table 1). There were no significant differences regarding the 3 separated periods (i.e. before 1990, between 1990 and 2000, and after 2000) of management of BD according to the 3 ethnics groups.

#### Survival rate according to ethnic origin

The 5-, 10- and 15 year survival rate was of 90%, 87% and 81% in sub Saharan African BD patients as compared to 96%, 93% and 91% and 99%, 96% and 94% in those from North Africa and Europe (p = 0.015), respectively (Figure 1).

**Figure 1:**
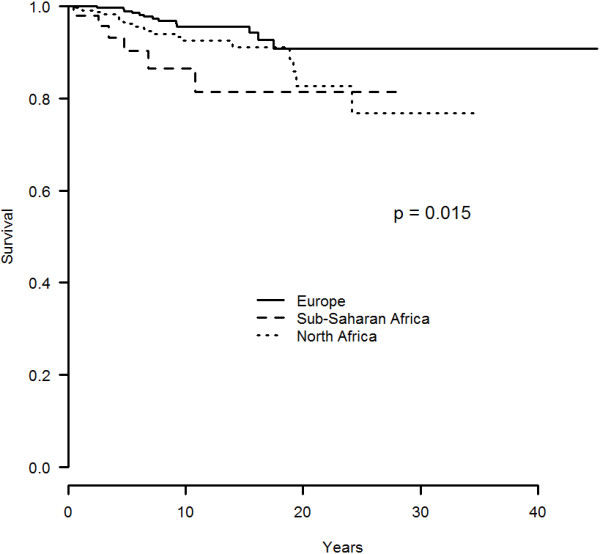
**Survival curve of 679 patients with BD according to their ethnic origin (Europe vs sub-saharan Africa vs North Africa).** BD, Behçet’s disease.

#### Factors associated with mortality

Comparison between alive and deceased patients showed a higher proportion of men and of cardiovascular involvement and a lower frequency of HLA B5 genotype in the deceased group (Table 2). The analysis of factors associated with mortality is summarized in Table 3. In univariate analysis, there was no significant association between mortality and oral ulceration, articular involvement, central nervous system involvement, and eyes involvement. Patients who died were younger (p = 0.002), more frequently of male gender (p = 0.001), had a higher frequency of cardiovascular involvement (p = 0.009), had more frequently glucocorticosteroids (p = 0.013) and immunosuppressants use (p = 0.020), more frequently originated from sub Saharan Africa (p = 0.015) and had a lower frequency of genital ulceration (p = 0.006). In multivariate analysis (Table 3), male gender (OR: 5.01, CI: 1.51-16.65), cardiovascular involvement (OR: 2.24, CI: 1.15-4.36) and African sub-Saharan origin (OR: 2.62, CI: 0.98-6.97) were independently associated with mortality.

**Table 2:** Comparative analysis between alive and deceased BD patients

**Parameters**	**N**	**Alive**	**N**	**Deceased**
	**729**	**(n = 729)**	**40**	**(n = 40)**
Age at diagnosis	724	30.6 [24.6; 37.19]	40	32.42 [27.0; 44.19]
Male sex	468	64.2%	37	92.5%
Genital ulcerations	448	61.45%	17	42.5%
Oral ulcerations	724	99.31%	40	100%
Articular involvement	354	48.83%	20	50%
Ocular involvement	482	66.21%	25	62.5%
CNS involvement	229	31.67%	15	37.5%
Cardiovascular involvement	301	41.29%	27	67.5%
Immunosuppressants	380	52.13%	30	75%
Corticosteroids	475	65.16%	35	87.5%
Anticoagulation	46	6.31%	3	7.5%
HLAB5*	281/538	52.23%	11/30	36.67%
Ethnicity				
Europe	356	48.83%	13	32.5%
Sub Saharan Africa	44	6.04%	6	15%
North Africa	329	45.13%	21	52.5%

CNS, central nervous system, BD, Behçet's disease.

*HLAB5 was available for 538 out of 729 patients.

Except where indicated otherwise values are the median, IQR.

**Table 3:** Factors associated with mortality in Behçet’s disease

	**Univariate analysis**		**Multivariate analysis**	
**Parameters**	**HR (CI 95%)**	***p***	**HR (CI 95%)**	***P***
Age at diagnosis	1.04 (1.01-1.07)	0.002	1.05 (1.02-1.08)	0.0007
Male gender	6.87 (2.1-22.3)	0.001	5.01 (1.51-16.65)	0.0085
Ethnic origin				
Europe	1	0.015		
North Africa	1.93 (0.96-3.85)			
Sub Saharan Africa	3.75 (1.43-9.88)		2.62 (0.98-6.97)	0.015
HLA B5	0.54 (0.26-1.13)	0.10		
Oral ulcerations	0.54 (0.1-2.2)	0.39	0.41 (0.22-0.78)	0.0069
Genital ulcerations	0.42 (0.22-0.79)	0.006		
Ocular involvement	0.81 (0.43-1.54)	0.53		
CNS involvement	1.05 (0.55-2)	0.88		
Articular involvement	0.8 (0.43-1.54)	0.49		
Cardiovascular involvement	2.43 (1.25-4.7)	0.009	2.24 (1.15-4.36)	0.0184
Corticosteroids	3.3 (1.29-8.43)	0.013		
Immunosuppressants	2.35 (1.15-4.8)	0.020		

CNS, central nervous system, BD, Behçet's disease.

#### Literature review

We found 11 manuscripts, with a total of 910 patients that assessed ethnicity related phenotypic or prognosis differences in BD [6-16]. Three of them [6,9,12] were excluded because two compared their patients’ characteristics to data reported in the literature, and one was using the same cohort of BD patients previously reported. We analysed 8 studies (783 patients), over a period of 1988 to 2012, that have addressed ethnicity related differences according to phenotype and outcome of BD [7,8,10,11,13-16]. The Table 4 summarized the main characteristics and conclusions of studies analysing ethnicity related differences in BD.

**Table 4:** **Literature review of studies that have addressed ethnycity related differences according to phenotype and outcome of BD **[7,8,10,11,13-16]

**First author**	**Country**	**Year of publication**	**Nb of patients**	**Ethnicity**	Observations*
Wechsler	Paris (France)	1988	196	French Men (n = 36) and North African men (n = 160).	No significant difference
Zouboulis	Allemangne	1997	196	Allemands (n = 82), Immigrés Turques (n = 86) patients originaires de pays étrangers autres (n = 28)	Plus d’atteintes oculaires chez les patients du Sud-Est de l'Europe (Italie, Gèce) et de Turquie. 25% des patients avec évolution défavorable, 3 décès, tous Allemands.
Zouboulis	Germany	1997	196	German (n = 82), Turkish immigrants (n = 86), and patients from other foreign countries (n = 28)	Ocular disease is more frequent in South-Eastern European patients and in Turkish immigrants.
Muhaya	Kurume (Japan) London (England)	2000	54	Japanese (n = 35) and British (n = 19) (including: 12 caucasians, 5 Middle Eastern, 1 African, 1 Asian)	Kurume patients have more active anterior uveitis and more posterior uveitis than London patients.
Krause	Tel Aviv (Israel)	2001	100	Jewish patients (n = 66) (most of them originated from Iran/Iraq, Turkey and North African countries) and Arabic patients (n = 34)	Arabic patients have more severe ocular diseases. Jewish patients from North African countries have higher disease severity score.
Kotter	Tübingen (Germany)	2004	65	German (n = 32) and Turkish descents (n = 33)	No significant difference
Rozenbaum	Northern area of Israel	2007	53	Arabs (n = 30) and Druzes (n = 23)	Higher frequency of uveitis, of deep vein thrombosis, and of CNS involvement, and a higher global severity score in Arabs.
Mahr	Seine-Saint-Denis County (France)	2008	79	European patients (n = 19) and non-European patients (n = 60).	No significant difference
Mohammad	Skåne (Sweden)	2012	40	Swedish ancestry (n = 12) and non-Swedish ancestry (28/40, 70%) [Middle East (n = 15), Africa (n = 2), East Asia (n = 2); Turkey (n = 2), Central and Eastern Europe (n = 6)]	No significant difference

*Difference with respect to phenotype or outcome of BD. CNS, central nervous system.

### Discussion

Ethnic origin is one of the factors that may modulate the prevalence and expression of BD. Analysis of the literature showed that studies assessing ethnicity related differences in BD are scarce and often derived from small samples thus preventing clear conclusions. Herein, we reported ethnicity-related differences with respect to phenotype and prognosis of BD in a French multiethnic country.

The comparison of our BD patients according to their ethnic origin showed a higher frequency of male in sub Saharan and North African patients as compared to those from Europe. Similar to variations in the clinical manifestations, gender distribution in BD patients varies widely depends on their ethnic origin and country of residence. For instance, the percentage of male BD patients in a recent study ranges from 27% in USA to 87% in Azerbaijan [17]. Male gender is a main factor associated with mortality in BD [18,19]. In our previous study, 92.7% of the BD patients who died were of male gender [19]. In multivariate analysis, male gender increased by 5 times the odds of death. Male gender and a younger age at onset have been previously reported to markedly influenced disease expression and course of BD [18]. Lastly, male patients tended to have more flare of BD compared to female [19].

We observed ethnicity-related differences regarding phenotype of BD. Our study shows a 1.6 times higher frequency of CNS involvement among patients from sub Saharan Africa as compared to those from North Africa and Europe. Cardiovacular involvement tended to be more frequent in sub-Saharan African patients but difference did not reach statistical significance. Arterial and cardiac complications are less common than venous lesions in BD, occurring in 1 to 7% of patients [20]. The concept of vasculo-Behçet’s has been adopted for cases in which vascular complications are present and often dominate the clinical feature that fit with the phenotype of our sub Saharan African BD patients. The main causes of death in BD included major vessel disease, and central nervous system involvement [18,19]. The frequency of CNS involvement in BD varies widely according country and ranges from 3% in Iran to 34% in Saudi Arabia [17]. The 48% rate of CNS lesions found in our sub Saharan African BD patients was clearly higher than the frequency observed in Saudi Arabia. Although not independently associated with mortality, CNS involvement accounted for 12% of deaths in our previous study [19]. In large studies addressing neurologic disease of BD, the mortality rate range between 5.5 and 20% [21,22]. The median period until death was of 4 years after neurological onset [21]. It was previously reported that BD has diverse clinical expression in various geographical areas. The pathergy reaction is considered highly sensitive and specific for BD in patients from Turkey and Japan, yet is frequently negative in patients from Western countries [23], or gastrointestinal (GI) involvement, which occurs in about one-third of patients from Japan, but rarely in Mediterranean countries. O’Neill et al. [24], described regional differences regarding several clinical manifestations of BD. They reported that BD patients from Middle Eastern countries and the Mediterranean basin generally have less widespread disease compared with patients from Western countries (i.e. UK and USA), manifested by lower rates of arthritis, vascular problems, and CNS abnormalities [24].

The HLA B5 allele was two times less frequently found in sub Saharan African BD patients as compared to those from Europe and North Africa and with the overall prevalence of HLA B5 allele usually reported in BD studies [25]. Large association analysis studies have reported that HLA B51 is carried by one-to two-thirds of BD patients and increases the risk of BD development by 6 [25]. Authors have suggested that HLA B51 positive and negative BD patients differed in that the former more frequently developed eye involvement [26] or sometimes CNS involvement [1] and the latter more commonly thrombophlebitis. However, based on two recent large meta-analyses, it has been shown that no real association exists between HLA-B51 positivity and the frequency of CNS involvement [25]. In contrast, uveitis, genital ulcerations and skin lesions were more frequent in BD patients carrying this allele. However, whether BD carriers of HLA-B51 allele exhibit a more severe disease course is still unknown.

The most original finding of our study is that sub Saharan African BD patients had up to 3 times higher mortality compared to North African and European patients. Their 15-year mortality rate was of 20% which was much higher than the 5-10% usually found in large series of BD patients [18,19]. The sub Saharan African group was independently associated with a poor outcome as well as male gender and cardiovascular involvement in our BD patients. These data has, to the best of our knowledge, never been described in the literature. We suggest that a particular attention should be given to sub Saharan African patients in BD. Krause *et al*. [8] have described the influence of ethnic origin on clinical expression and disease severity in Israeli patients. They studied 100 patients fulfilling International Study Group criteria for BD including 66 Jewish and 34 Arabic patients. The 3 largest ethnic groups of Jewish patients were from Iran/Iraq (n = 21), Turkey (n = 12), and North Africa (n = 21) countries. Arabic patients had more severe ocular disease with significantly higher rate of posterior uveitis (20.6 vs 4.6%). In the 3 most common Jewish ethnic groups, patients form Iran/Iraq disclosed higher rate of folliculitis (61.9 vs and 28.6%). Jewish patients from North African countries had higher rate of ocular disease and disease severity score was significantly higher in this population. Zouboulis *et al.*[16], analysed the clinical features of 196 BD patients [German (n = 82), Turkish immigrants (n = 86) or immigrants from other countries (n = 28)]. They found a higher rate of ocular disease in South-Eastern European patients (Italy and Greece) compared to South-Western and North European patients. Frequency of ocular lesions was also more frequent in Turkish patients compared to German.

We acknowledge some limitations in the current study. Our analysis was performed as a retrospective review. Socioeconomic differences may exist in our study population, especially between European and African (i.e. North or sub Saharan) patients. However, the time between first symptoms to BD diagnosis was equivalent between groups. Access to medical system does not seem to significantly account for the differences observed because the French social security system covers all medical care. One can hypothesize that genetic factors (i.e. HLA B 51 and/or others) may be involved in ethnic differences with respect to phenotype and outcome of BD. We were not able to collect country of birth of BD patients in a comprehensive manner in order to provide some insight into environment versus genetic influences.

In conclusion, in a French multiethnic country, sub Saharan African BD patients exhibited a worse prognosis. They displayed a higher frequency of CNS involvement and trend toward higher cardiovascular involvement compared to BD patients from Europe or North Africa. Their 15-year mortality rate reached 20% which is 3 times higher than the overall mortality in BD. Taken together, these data suggest that a particular attention should be given to sub Saharan African BD patients.

## Competing interests

The authors declare that they have no competing interests.

## Authors’ contributions

LS and DS wrote the paper. DS designed the study. MRR performed the statistical analysis. LS, BW, CC, JCP, PC and DS followed the patients. All authors read and approved the final manuscript.
